# A Rare Cause of Cavitary Lesion in the Lung: Richter's Transformation

**DOI:** 10.1155/2015/945268

**Published:** 2015-02-24

**Authors:** Fatma Yıldırım, İskender Kara, Sedat Yıldız, Nalan Akyürek, Kadir Acar, Melda Türkoglu, Gülbin Aygencel

**Affiliations:** ^1^Department of Pulmonary Medicine, Gazi University Faculty of Medicine, Ankara, Turkey; ^2^Department of Anesthesiology and Reanimation, Gazi University Faculty of Medicine, Ankara, Turkey; ^3^Intensive Care Unite, Department of Internal Medicine, Gazi University Faculty of Medicine, Ankara, Turkey; ^4^Department of Pathology, Gazi University Faculty of Medicine, Ankara, Turkey; ^5^Department of Hematology, Gazi University Faculty of Medicine, Ankara, Turkey

## Abstract

Richter's transformation (RT) refers to the development of aggressive lymphoma during the course of CLL. Clinically, patients with RT present with an aggressive disease course with rapidly enlarging lymph nodes, hepatosplenomegaly, and elevated serum lactate dehydrogenase levels. But rarely it presents with extra nodal organ involvement at the beginning. Common sites of extra nodal involvement are the gastrointestinal tract, eye, central nervous system, lung, and kidney. We are reporting this case that was presented with RT in the lung involvement diagnosed while researching cavitary lesion etiology.

## 1. Introduction

Richter's transformation (RT) is the development of high grade lymphoma, especially diffuse large cell B lymphoma in cases with chronic lymphocytic leukemia (CLL) [1]. The incidence of this transformation is 1–10% [2]. It begins most commonly as lymph node involvement, but it can also begin as extranodally such as gastrointestinal system, skin, nervous system, and lung involvements [3].

It is necessary to develop an immunophenotypic shift for the transformation of chronic lymphocytic leukemia to RT; however, it is not known in which patient and when it develops. Furthermore, it is also known that the prognosis is poor in patients with RT [3, 4].

In the present case, the cavitary lesions in the lung were demonstrated to be caused by lymphoma in a patient who had been followed up with the diagnosis of CLL and the patient was accepted to have RT. The presence of cavitary nodular lesions in the lungs is a very rare condition at the diagnosis time of RT, and, as such, the case was presented.

## 2. Case

An 80-year-old female patient was admitted to our hospital with weakness. Her peripheral blood counts were as follows: white blood cell-24 × 10^9^/L; hemoglobin (Hb)-11.9 g/dL; platelet count-204 × 10^9^/L. Peripheral smear showed absolute lymphocytosis with mature morphology (neutrophils 22%, lymphocytes 64%, eosinophils 7%, basophils 0%, and monocytes 7%, resp.). Bone marrow aspiration revealed the presence of similar small mature lymphocytes accounting for 30% of nucleated cells. Immunohistochemical staining revealed that the lymphoid cells were positive for CD20 and CD23 and these cells were negative for CD10, cyclin D1, TDT, CD3, and CD5. Overall pattern was consistent with B-cell prolymphocytic leukemia. The patient had been followed up with the diagnosis of CLL for two years without any medication.

Two years later, patient was accepted to our clinic with complaints of malaise, fatigue, loss of appetite, weight loss of 10 kg within the last three months, and cough, sputum production, fever, and deterioration in her state of consciousness within the last month. Upon her initial physical examination, she was confused and had tachypnea and tachycardia. On her lung examination the breath sounds were decreased in the basal parts of lungs. There was splenomegaly in the abdominal examination. Peripheral lymphadenopathy was not detected.

**Figure 1:**
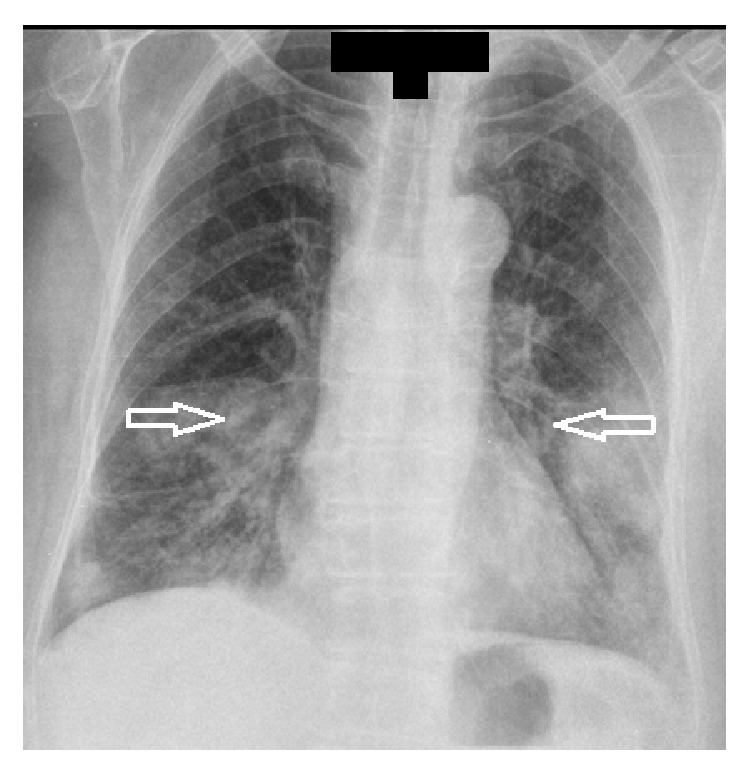
Posteroanterior chest X-ray showing parenchymal infiltrations and cavitary lesions.

Her peripheral blood counts were as follows: white blood cell-13 × 10^9^/L; hemoglobin (Hb)-8.9 g/dL; platelet count-58 × 10^9^/L. In the biochemical analysis, hyperglycemia (glucose: 242 mg/dL) and high levels of lactate dehydrogenase (LDH) 412 U/L (normal range 0–248 U/L) were detected. On the posteroanterior chest X-ray, homogenous density increases in the lower zones and consolidation areas in the right lower zone and its vicinity were observed (Figure 1).

**Figure 2:**
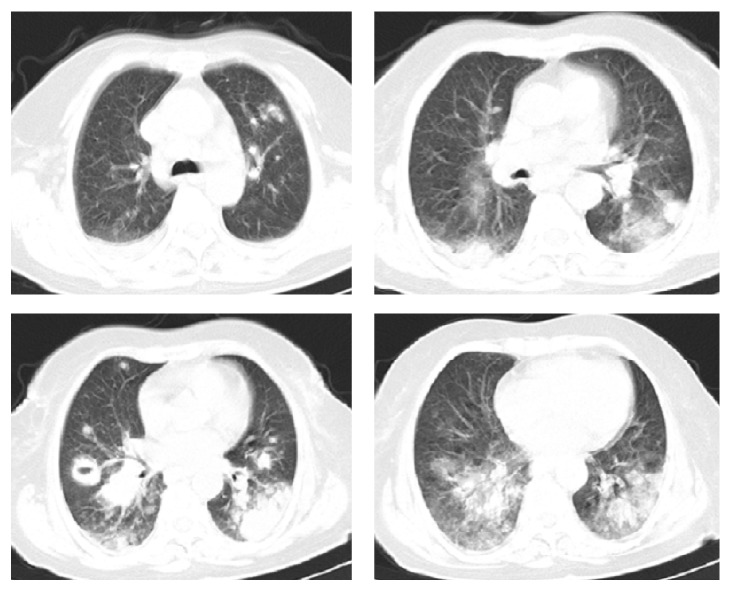
Parenchymal window of computed tomography scan showing parenchymal nodules, infiltrations, pleural masses, and cavitary lesion.

**Figure 3:**
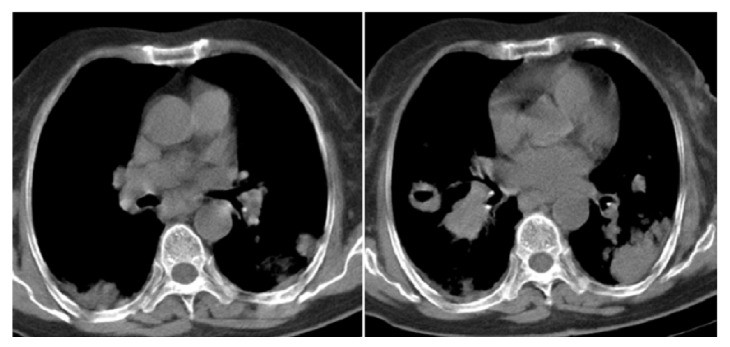
Mediastinal window of computed tomography scan showing bilateral mediastinal, hilar adenopathies, and cavitary lesions.

**Figure 4:**
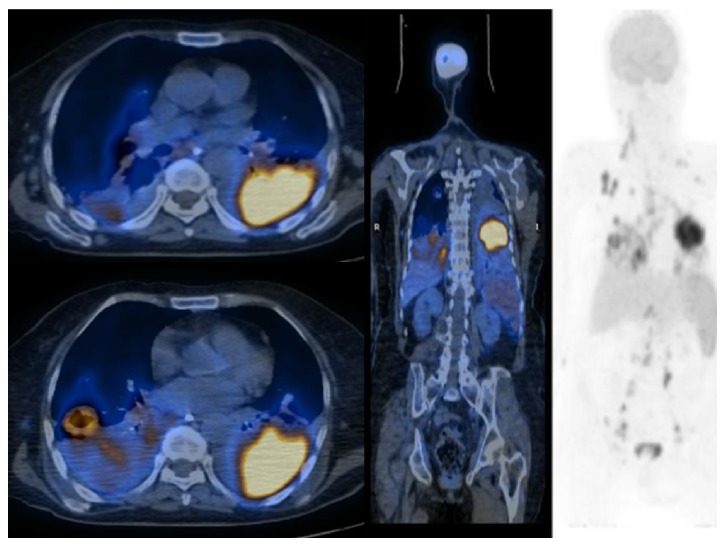
PET/CT scan showing increased 18F-FDG uptake multicompartmental lymphadenopathies and lung lesions.

**Figure 5:**
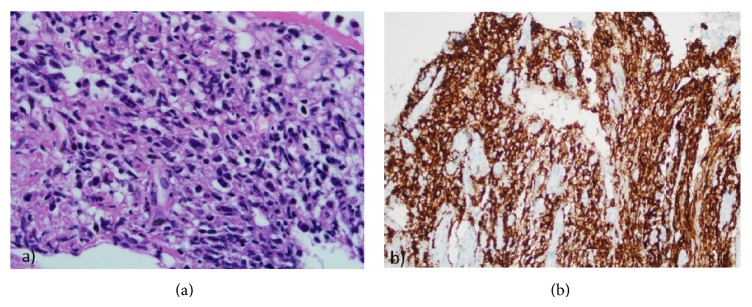
(a) Section shows an infiltrate of large atypical cells (H and E, ×400). (b) The neoplastic cells are diffusely CD20 positive (CD20 immunostain, ×400).

**Figure 6:**
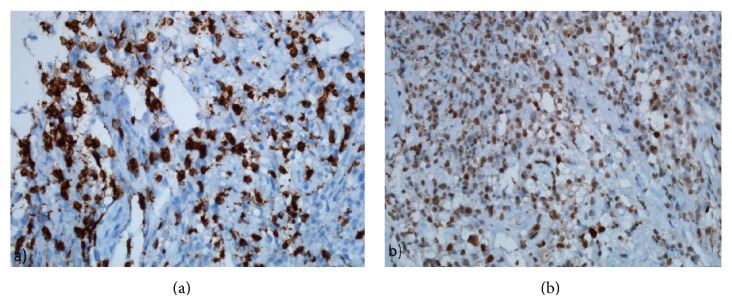
(a) The neoplastic B lymphoid cells also show diffuse MUM-1 positivity (MUM-1 immunostain, ×400). (b) Ki-67 index was 80%.

**Figure 7:**
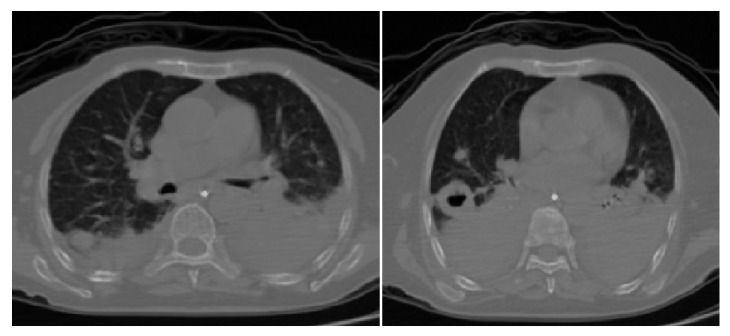
Parenchymal window of computed tomography scan showing bilateral pleural masses, effusions, and enlarged cavitary lesion at the time of respiratory insufficiency.

Cranial computerized tomography (CT) was taken, as the patient was confused and thorax CT was taken due to the lesions in the chest X-ray. The cranial CT of the patient was interpreted as normal. On thorax CT, a cavitary lesion measuring 2.9 × 3.5 × 3.1 cm was detected in the superior segment of the right lower lobe, diffuse multiple nodular lesions in lungs, and multiple mediastinal and bilateral hilar lymphadenopathies, the largest measuring 1.5 cm in diameter, were detected (Figures 2 and 3). Diagnostic bronchoscopy was performed. Acid resistant basil (ARB) from bronchoalveolar lavage was researched; cultures and polymerase chain reaction (PCR) were done in terms of tuberculosis, candida, and other infections. Cytology, transbronchial biopsy from the cavitary lesion in the superior segment of the lower lobe of the right lung, and mucosal biopsies were obtained, and a pathological examination was sent. The results of the cultures, ARB, and PCR were negative. After infectious causes were ruled out, PET/CT was obtained. On PET/CT, bilateral cervical, supraclavicular, axillary, mediastinal, and abdominal lymph nodes with SUVmax involvement between 4.5 and 10.5 were detected; SUVmax 10.3 involvement was detected in the cavitary lesion in the superior segment of the lower lobe of the right lung. SUVmax 19.1 involvement was detected in a consolidated mass in the lower lobe of the left lung (Figure 4). In the pathological examination of the transbronchial biopsy from cavitary lesion wall, infiltration composed of large, atypical lymphoid cells, which formed scattered, small groups among the small lymphoid cells were observed. Atypical lymphoid cells made up less than 10% of T cells in the ground. In the immunohistochemical staining, CD20, MUM-1, CD30, and PAX5 were positive in the atypical lymphoid cells. Staining with CD3 and CD5 in small, mature T lymphocytes was observed. The diagnosis was consistent with “T cell rich, large B-cell lymphoma” (Figures 5 and 6). The patient was examined with the hematology department. Due to the patient's poor condition, chemotherapy containing only steroids and rituximab was planned. During the follow-up, the patient had respiratory failure and she was intubated and transferred to the intensive care unit (ICU) (Figure 7). The patient died on the seventh day of ICU follow-up.

## 3. Discussion

Richter's transformation (Richter syndrome-RT) was used for the first time in 1928 by Maurice Richter, to define an aggressive, large cell lymphoma that developed from CLL. The incidence of RT is approximately 1–10% [1–3]. There are no risk factors defined for this transformation. RT generally develops within 2–4 years, independent from the disease duration, stage of the disease, treatment, and response to treatment [3, 4]. In the current case, RT developed on the basis of CLL at the end of two years, when the disease had a mild course and the patient was under follow-up without treatment.

Richter's transformation generally appears with symptoms of fever, weight loss, hepatosplenomegaly, and peripheral lymphadenopathy. Anemia, neutropenia, thrombocytopenia, and increased LDH levels are important laboratory findings. It is generally observed initially in the lymph nodes and bone marrow. However, the disease might appear as extranodal involvement, such as in the gastrointestinal system, eyes, testicles, central nervous system, lung, kidney, and skin [5, 6]. In the current case, RT appeared with symptoms including fever, loss of appetite, and laboratory findings including anemia, thrombocytopenia, high LDH levels, and extranodal involvement (lung).

Positron emission tomography (PET/CT) is recommended to demonstrate the transformation from CLL to lymphoma and to determine the exact localization for biopsy to confirm the pathological diagnosis. PET/CT could also be used to demonstrate the extranodal involvement, in the determination of the invasiveness of the disease and in an assessment of the response following treatment [7, 8]. In the current patient, PET/CT was conducted and it was observed that the involvement was highest in lung lesions (extranodal involvement).

Chemotherapy (cyclophosphamide, vincristine, prednisolone, methotrexate, doxorubicin, and cytarabine containing regimes), monoclonal antibodies (rituximab), and stem cell transplantation could be used in the treatment of RT. The rate of response to treatment is between 27 and 48% [9]. Although the treatment regimes are aggressive, the expected median duration of life is 5–8 months [10–12]. In the current study, aggressive chemotherapy could not be administered due to the advanced age and poor performance, and the patient died within the hospitalization period.

Lung involvement could be observed in lymphoma. Pulmonary lymphoma is more common with Hodgkin's lymphoma (HL) than with non-Hodgkin's lymphoma (NHL). Lung involvement is usually associated with mediastinal nodal disease in HL; NHL can present as lung disease alone. Parenchymal involvement in HL is seen in up to 11.6% of the patients. Morphologically, lymphoma can present with a whole spectrum of changes ranging from nodules, mass lesions with or without cavity, endobronchial mass, ground glass opacities, a reticular interstitial pattern with involvement of the interstitial lymphatic system, or consolidation with an air bronchogram [13–15]. In the current case, NHL developed on the basis of CLL by RT and presented itself as parenchymal cavitary nodular lesions, which are observed in the lung in particular. In this respect, the present case is an unusual case.

In conclusion, it should be kept in mind that cavitary nodular lesions in the lung could be observed when RT develops in patients with CLL.
